# Natural interfaces and virtual environments for the acquisition of street crossing and path following skills in adults with Autism Spectrum Disorders: a feasibility study

**DOI:** 10.1186/s12984-015-0010-z

**Published:** 2015-02-19

**Authors:** Mario Saiano, Laura Pellegrino, Maura Casadio, Susanna Summa, Eleonora Garbarino, Valentina Rossi, Daniela Dall’Agata, Vittorio Sanguineti

**Affiliations:** Department Informatics, Bioengineering, Robotics and Systems Engineering, University of Genoa, Via Opera Pia 13, 16145 Genoa, Italy; Department Primary Care, ASL3 Genovese, Genoa, Italy

**Keywords:** Natural interfaces, Virtual environments, Kinect, Autism spectrum disorder, Safety skills, Adults

## Abstract

**Background:**

Lack of social skills and/or a reduced ability to determine when to use them are common symptoms of Autism Spectrum Disorder (ASD). Here we examine whether an integrated approach based on virtual environments and natural interfaces is effective in teaching safety skills in adults with ASD. We specifically focus on pedestrian skills, namely street crossing with or without traffic lights, and following road signs.

**Methods:**

Seven adults with ASD explored a virtual environment (VE) representing a city (buildings, sidewalks, streets, squares), which was continuously displayed on a wide screen. A markerless motion capture device recorded the subjects’ movements, which were translated into control commands for the VE according to a predefined vocabulary of gestures. The treatment protocol consisted of ten 45-minutes sessions (1 session/week). During a familiarization phase, the participants practiced the vocabulary of gestures. In a subsequent training phase, participants had to follow road signs (to either a police station or a pharmacy) and to cross streets with and without traffic lights. We assessed the performance in both street crossing (number and type of errors) and navigation (walking speed, path length and ability to turn without stopping).

To assess their understanding of the practiced skill, before and after treatment subjects had to answer a test questionnaire. To assess transfer of the learned skill to real-life situations, another specific questionnaire was separately administered to both parents/legal guardians and the subjects’ personal caregivers.

**Results:**

One subject did not complete the familiarization phase because of problems with depth perception. The six subjects who completed the protocol easily learned the simple body gestures required to interact with the VE. Over sessions they significantly improved their navigation performance, but did not significantly reduce the errors made in street crossing. In the test questionnaire they exhibited no significant reduction in the number of errors.

However, both parents and caregivers reported a significant improvement in the subjects’ street crossing performance. Their answers were also highly consistent, thus pointing at a significant transfer to real-life behaviors.

**Conclusions:**

Rehabilitation of adults with ASD mainly focuses on educational interventions that have an impact in their quality of life, which includes safety skills. Our results confirm that interaction with VEs may be effective in facilitating the acquisition of these skills.

**Electronic supplementary material:**

The online version of this article (doi:10.1186/s12984-015-0010-z) contains supplementary material, which is available to authorized users.

## Background

Learning how to move in the surrounding space is an important stage in the development of any living being. The ability of crossing a street safely and autonomously is crucial for the protection of oneself and of others and is essential for personal autonomy [1]. Street crossing represents one of the most widespread dangers for children, adolescents and elderly people, both with and without disabilities [2]. Persons with cognitive or motor deficits are particularly at risk, and often need assistance when walking in outdoor environments.

Autism Spectrum Disorders (ASDs) are a class of complex neurobehavioral conditions, characterized by an impairment of social interaction, verbal and non-verbal communication, repetitive and stereotypical forms of behavior, and rigidity in the habits [3]. ASD symptoms appear during the first three years of life and may vary over time. Lack of social skills and a reduced ability to determine when to use these skills also contribute to the overall disability. Some manifestations of autism include delays in cognitive development, language, gestures and movements, in the capacity of imagination, in symbolic play and in recognizing emotions; presence of sensory hypersensitivity [3], delays in executive functions [4] and in learning how to conduct crucial activities of daily living. Only a small fraction of these persons are able to live and work independently in adulthood [5].

Although no specific data are available for ASD, adults with developmental disability have been reported to have a higher risk of fatal accidents when crossing a street [6,7]. These risks can be mitigated through educational interventions.

In the case of persons with ASD, interventions should specifically address the development of skills that are crucial for an independent and autonomous life, such as the use of public transportation, the ability to follow signs and to head for a place, and to cross a road safely.

Several methods have been developed to teach these abilities, ranging from educational videos and board games; classroom activities based on model street intersections and a doll; and practice in protected street environments [7].

Virtual Reality (VR) is a combination of technologies to support the creation and exploration of 3D computer-generated representations of environments with a realistic appearance (Virtual Environments, VEs). VEs could be ideal platforms for teaching social understanding and safety skills to persons with ASD [8,9]. Interaction with VEs provides a safe and controlled environment to enable realistic simulations of real-life situations. VEs may provide experiences that can help patients to understand concepts as well as to learn how to perform specific tasks, which can be repeated as often as needed [10]. For example, interacting with a virtual replica of public places (cafes, supermarket, road and public transportation) may improve the ability to recognize specific situations (presence of cars, prohibition of walking into flowerbeds) and to perform typical daily life tasks (taking a bus or getting a coffee). Further, the realism of simulated environments may increase the chances to transfer the learned skills into their everyday life. In the case of ASD, VEs may be even better suited for learning than real environments, as they allow (i) to remove competing and confusing stimuli from the social and environmental context and to add them again as learning progresses; and (ii) to manipulate time using short breaks to clarify to the participants the variables involved in the interaction process; see [11] for a review. Moreover VEs allow subjects to learn by playing [12]. In [13], two low-functioning children with autism were reported to track events with appropriate orienting movements when experiencing a fully immersive VE. Interaction with a VE has been proven effective to facilitate the acquisition of social skills used in daily life activities, like using public transportation [14] and visiting a café [15]. In all cases, ASD subjects were reported to improve their social skills after the VE sessions. VR games were used to engage ASD children in symbolic play [16]. The results showed a significant advance in pretend play abilities, and a high degree of generalization of the acquired teaching. Other studies used avatars in multi-user VEs to mediate communication between patient and therapist, to examine and investigate the ability to recognize emotions [17], to teach how to manifest their emotions and to understand those of other people [18].

Several studies have addressed the use of VR to teach persons with ASD pedestrian skills, like following a path with signs [19] and like street crossing [20,21]. Similar approaches have been used to teach safe street crossing skills to stroke survivors with unilateral spatial neglect [22], and to both children and adults with no disability [23-25]. In summary, while several studies target healthy persons - children, adolescents or adults - fewer studies focus on children and adolescents with ASD, and no studies so far have specifically targeted the adult ASD population.

Interaction with VEs can be mediated by sensors and displays such as mouse, keyboard, and computer screens. The recent introduction of goggles and helmets has enabled more immersive forms of interaction, but these devices may lead to sensory over-stimulation, thus possibly inducing discomfort and general malaise phenomena, e.g. headache, dizziness, nausea [26]. These effects may be magnified in individuals with ASD, who tend to have a greater perceptual sensitivity [27].

In comparison to simpler interfaces (e.g. mouse + keyboard, or gamepad), gesture-based interfaces (Natural Interfaces, NI) – e.g. Nintendo Wii and Microsoft Kinect - may improve the interaction with the VE [28] while mitigating the sensory overstimulation problems as they do not require sensors or devices in direct contact with the body of the user. In fact, individuals with ASD have been reported to easily accept to use natural interfaces (NI) and body movements to control the VEs [29,30]. The use of NIs might also improve the efficacy of interaction, by increasing the awareness of their own body and/or by improving the generalization of the competences acquired in the VE to real-life situations.

The purpose of this study is to examine whether an integrated approach based on virtual environments and natural interfaces is effective in teaching safety skills in adults with ASD. We specifically focus on pedestrian crossing with or without traffic lights, following signs to go to a place (police or pharmacy).

## Methods

### Subjects

The study involved seven subjects of age 29 ± 10 years (range: 19–44), all male, recruited among the outpatients of the Department of Primary Care of the Local Health Authority of the city of Genoa (ASL3 Genovese). They all had a diagnosis of ASD according to the DSM-V criteria [3], in most cases made in their infancy, and further confirmed by one of the authors (E.G.) – a trained neuropsychiatrist. The exclusion criteria were: (i) presence of motor symptoms that could affect the translation of movements into VE interaction commands; (ii) severe hypovision (visual acuity with corrective lenses less than 1/10); (iii) inability to discriminate the colors of traffic lights and to understand street signs; and (iv) aggressive conduct. We also excluded persons whose stereotypical movements (e.g. repeated banging of hands, back and forth rocking movements) were so severe that they could affect the translation of their movements into VE navigation commands. We did not explicitly exclude subjects with stereotypical behaviors of verbal type. A trained therapist assessed all the above conditions.

All participants had similar initial levels of pedestrian skills. Their parents all reported that they did not trust them to go out alone, and they only went out if accompanied by a caregiver.

For each subject, an experienced neuropsychologist assessed disease severity according to the criteria laid out by the DSM-V manual [3] and the intelligence quotient (IQ) through the Wechsler Adult Intelligence Scale-Revised (WAIS-R) [31], adapted for Italy [32].

Table 1 summarizes the subjects’ demographic information. The IQ score is reported separately for the WAIS-R’s verbal (verbal IQ, VIQ: information, comprehension, arithmetic, digit span, similarities and vocabulary) and performance sections (PIQ: picture arrangement, picture completion, block design, object assembly and digit symbol). We also report the comprehensive full-scale IQ (FSIQ).

**Table 1:** **Subjects demographics**

**Subject**	**Sex**	**Age**	**Severity**	**VIQ**	**PIQ**	**FSIQ**	**Speech**	**ID**
S1	M	24	1	92	103	97	Y	N
S2	M	29	2	NA	55	NA	N	Y
S3	M	44	2	55	68	56	Y	Y
S4	M	21	2	NA	95	NA	N	N
S5	M	19	2	NA	98	NA	N	N
S6	M	23	1	80	90	82	Y	N
S7*	M	40	3	54	72	57	Y	Y
Mean ± SD	-	29 ± 10		40 ± 40	83 ± 18	42 ± 41	-	-

Severity level is expressed in a 3-point scale (1: minimum; 3: maximum).

The IQ score is expressed as verbal (VIQ), performance (PIQ) and full scale (FSIQ).

Speech: ability to speak (Y/N).

ID: intellectual disability (Y/N).

VIQ and FSIQ are not applicable (NA) in subjects that were unable to speak. In this case only PIQ is reported.

*S7 was unable to complete the full procedure.

Based on their WAIS-R score, we classified subjects in terms of presence or absence of speech and presence or absence of intellectual disability. Specifically, we defined subjects with a FSIQ (or PIQ in the case of absence of speech) <70 as with intellectual disability (ID). Subjects with FSIQ (or PIQ) >70 were considered as with no-ID. This classification is based on the informal high-functioning vs low-functioning definitions [33].

The research has been approved by the Ethical Committee of ASL3 “Genovese” and conforms to the ethical standards laid down in the 1964 Declaration of Helsinki that protects research subjects. Each subject’s parent or legal guardian signed a consent form in adherence to these guidelines.

### Experimental apparatus and virtual environment

**Figure 1:**
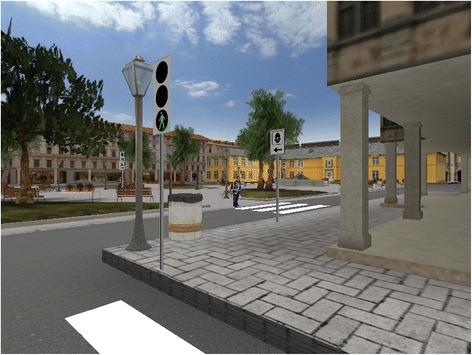
**Screenshot of the virtual environment used in the study.**

The experimental apparatus included a video projector, displaying a virtual reality environment on a 2 m × 2 m screen. The participants were required to stand in front of the screen, at a distance of approximately 2 m. The screen continuously displayed a realistic city environment, including buildings, sidewalks, streets, and squares; see Figure 1.

Visual rendering was based on a first-person perspective. The VE was based on the open-source virtual reality platform NeuroVR 2.0 [34]. To the standard ‘city’ environment provided by NeuroVR, we added traffic lights, pedestrian crossings, road signs, and a number of distractors (e.g. cars, other people, and dogs). All objects in the scene are static, but the traffic lights could switch to green, yellow, and red. Dogs barked when subjects approached them.

Instead of using mouse and keyboard to interact with the VE, we used a markerless motion capture device (Microsoft Kinect), placed below the screen to record the subjects’ full-body movements in 3D space. The device has limited accuracy [35], but allows reconstructing the trajectories of ‘virtual’ markers (e.g. head, right hand, left foot, etc.) in real-time, at a 30 Hz sampling rate.

In this experiment, the reconstructed trajectories of 20 virtual ‘markers’ were used to identify a dictionary of gestures, which were used to control the subject’s interaction with the VE. The dictionary of gestures is summarized in Table 2.

**Table 2:** **Dictionary of gestures**

**Body gesture**	**VE command**
Right foot forward/backward	Walk forward/backward
Left/right arm abduction	Turn left/right
Left/right torso rotation	Watch left/right

Body gestures were mapped into VE commands by using the Flexible Action and Articulated Skeleton Toolkit (FAAST) [36], a middleware specifically designed to facilitate Kinect-based, full-body control of games and VR applications.

### Task and training protocol

The study protocol consisted of a total of 10 sessions (1 session/week) and involved a familiarization (sessions 1–5) and a training (sessions 7–9) phase. Before the beginning and after the end of the training phase, we ran two assessment sessions (sessions 6 and 10).

The familiarization phase had the purpose of practicing the vocabulary of gestures necessary to interact with the VE. Under the supervision of a therapist, subjects interacted with the standard ‘city’ environment provided by NeuroVR for a maximum of 30 minutes per session. This is a simplified environment, providing neither specific cues (signs, crosswalks, traffic lights) nor specific directions (road signs). In a later phase we placed arrow signs on the floor, which the subjects were required to follow.

During familiarization, a therapist observed the participants’ behavior and showed them the dictionary of gestures that are necessary to interact with the VE. Specifically, the therapist initially showed the subjects the movements to make in order to control the VE. Wherever necessary, he/she guided the subject’s movements by pushing or pulling them. Finally, the subjects were required to repeat the same movements on their own, with the therapist only providing verbal cues.

**Figure 2:**
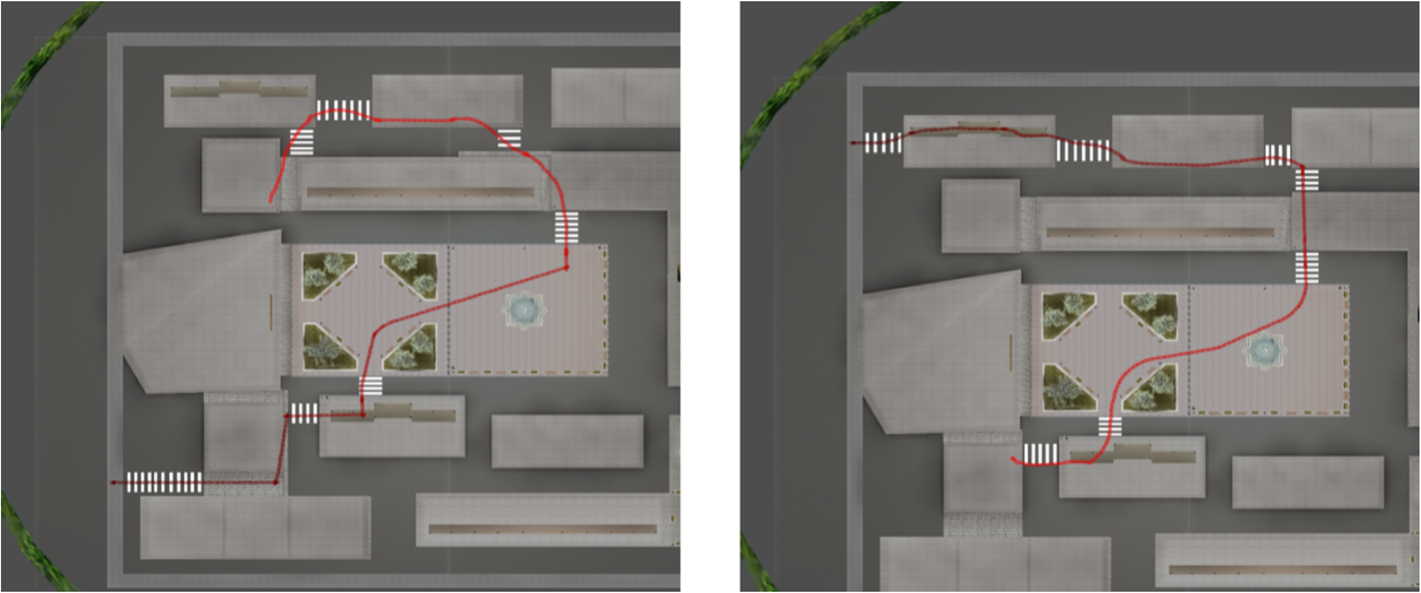
**The two different paths used in the training sessions: path A (left) and path B (right).** The red line indicates the minimum-length path.

The treatment phase consisted of three sessions (maximum duration: 45 min). During each session, the participants had to complete two different paths (A and B), by following arrows and signs. Path A led to a pharmacy, path B to a police station. Each path had exactly the same length and required them to cross at least seven roads, see Figure 2.

Paths A and B were presented alternately, for a total of three repetitions for each path. Subjects had to complete each path within a time limit of 10 minutes, then the software application switched to the next one.

Over sessions we gradually increased task difficulty. In the first session the path only included crosswalks, without traffic lights. Subjects had to stop and look at both sides of the street before crossing a road. In the second session, all crosswalks had traffic lights. In this case, subjects were also required to wait for the green light before crossing. In the third session, there were both types of crosswalks (with and without traffic lights) plus a number of distractors (other people, dogs, street noise). All errors - i.e. crossing without looking, crossing with red/yellow light, walking outside the sidewalk or crossing outside the crosswalk – automatically triggered an acoustic alarm.

During the treatment phase the therapist monitored the behavior of the participant and provided both verbal correction and reinforcement. For example, if a subject crossed the road when the traffic light was red or yellow, the therapist reminded him of the rules of conduct for this particular action.

During the assessment sessions – session 6 (PRE-treatment, T0) and session 10 (POST-treatment, T1) – subjects had to complete path B with the maximum difficulty level. During these sessions the therapist only observed and recorded the subjects’ behaviors, without commenting or providing suggestions.

To test whether the subjects transferred the learned behaviors outside the VE, we used two approaches. Before and after treatment, i.e. in session T0 and T1, subjects had to fill a questionnaire aimed at assessing their understanding of the practiced skill. Questions were presented both in writing and visually. We developed the subjects’ questionnaire specifically for this work, but layout and organization were derived from a questionnaire used in a previously published study [15]. We designed the questions to specifically assess the subjects’ knowledge of the rules for street safety.

The questionnaire presented a number of situations in both text and picture formats. For each situation there were two possible answers (actions to perform), presented as pictures. In this way all subjects could answer, irrespective of their reading and/or verbal skills. Note that not all subjects could speak, but all could either read or at least understand the situations when presented as pictures.

Parents/legal guardians were also required to complete a questionnaire. This had the objective to assess to what extent they considered that subjects had improved their behavior in real-life situations. To get a second opinion about skill transfer, we administered the same questionnaire to each subject’s personal caregiver. With a formal training as health education professionals, personal caregivers are responsible for each subject’s personalized rehabilitation program but were blind about this treatment. The questionnaires used for subjects, parents and caregivers are provided as Additional files 1 (subjects) and 2 (parents and caregivers).

### Data analysis

#### Outcome measures related to the VE

To assess street crossing performance we focused on three types of errors: (i) crossing without looking, (ii) crossing with red/yellow light, and (iii) walking outside the sidewalk or crosswalk. These errors were automatically recorded by the NeuroVR application. We also looked at the total number of errors (sum of all the above). As the different degrees of difficulty in the training sessions affect number and type of errors (e.g. there are no traffic lights on session 7 – first day of the treatment phase), we limited the error analysis to the pre-treatment and post-treatment sessions, where the experimental conditions were identical. We specifically asked whether there was a significant reduction in the number of errors between PRE (T0) and POST (T1) treatment.

We first tested the data for normality (Lilliefors’ test). We then tested the effect of treatment through a paired-samples t-test if the data normality was not rejected, and a Wilcoxon’s signed rank test otherwise.

The ability to follow street signs in order to reach the target destination is hard to quantify in terms of number of errors (for instance, whether the direction taken after each sign was correct/wrong) because it is hard to account for subsequent corrections. Instead, we took more ‘global’ measures of the quality of a given path. The NeuroVR application automatically records the subjects’ 2D position **x**(t) and the instantaneous movement orientation θ(t) within the VE, both at a 2 Hz sampling rate. From these quantities, for each path we computed the following indicators: average speed, path length, displacement (‘figural distance’) from the ‘optimal’ path (corresponding to 200 m length for both path A and B); and a ‘composition index’ (CI) between translational and rotational motion.

Average speed and path length were directly calculated from x(t). Figural distance (FD) is a measure of similarity between two trajectories A and B. By denoting as n_A_ and n_B,_ the lengths of the two trajectories, and defining as ${d}_{AB}(i)=mi{n}_j\left|\overrightarrow{x_A}(i)\mathit{\hbox{-}}\overrightarrow{x_B}(j)\right|$ the minimum distance between the i-th sample of trajectory A and the points of trajectory B – and, conversely, as *d*_*BA*_(*i*) the minimum distance of the i-th sample of trajectory B and the whole trajectory A, the figural distance (FD) between A and B is defined as [37]:

1 $$ F{D}_{AB} = \frac{1}{n_A+{n}_B}\left[{\displaystyle {\sum}_{i= 1}^{n_A}{d}_{AB}(i)+}{\displaystyle {\sum}_{i=1}^{n_B}{d}_{BA}(i)}\right] $$

We also looked at whether subjects were able to translate (walk) and rotate (turn) at the same time. From the NeuroVR recordings we calculated a composition index (CI), defined as the fraction of the movement path that simultaneously involved both translation and rotation - walking and turning:

2 $$ \mathrm{C}\mathrm{I}=\frac{{\displaystyle {\sum}_t}\left[\left(\omega (t) > {\omega}_{th}\right)\  AND\ \left(v(t) > {v}_{th}\right)\right]}{{\displaystyle {\sum}_t}\left[\left(\omega (t) > {\omega}_{th}\right)\  OR\ \left(v(t) > {v}_{th}\right)\right]} $$

Translational speed and angular velocity were calculated, respectively, as the rates of change of the norm of the displacement: v(t) = |Δx|/Δt and the rotation ω(t) = Δθ/Δt between two subsequent samples of x(t) and θ(t): Δx = x(t + Δt)-x(t), where Δt = 0.5 s is the sampling interval. A large CI indicates that the subject does not need to stop in order to take a turn; in other words, the subject can make two movements simultaneously (specifically, putting the right foot forward and abduction of the arm).

To test the overall effect of the treatment, we compared all indicators between the pre-treatment (T0) and the post-treatment (T1) assessment sessions. Even in this case, we first tested the data for normality. Then we tested the effect of treatment through either a paired-samples t-test or a Wilcoxon’s signed rank test.

Here and in all subsequent statistical procedures, we accepted p = 0.05 as the threshold for significance. We recognize that multiple tests increase probability of a Type 1 error; however, because the focus of this study is to explore how the treatment affects performance and not to assess outcomes of the treatment, we have chosen to err in that direction rather than applying the Bonferroni correction which would decrease the threshold for significance to 0.05/8 = 0.00625.

### Subjects’ questionnaire

To assess the acquisition of the street safety skills, we required all subjects to complete a questionnaire both before and after the treatment. The questionnaire involved a total of six yes/no questions, which were presented both in writing and visually; see Table 3 and Additional file 2. We used McNemar’s test to assess the changes in the errors made in each individual question. We also looked at the total number of errors (wrong answers) before and after treatment. Depending on the outcome of the normality test, we used either a paired-samples t-test or a Wilcoxon’s signed-rank test for matched pairs.

**Table 3:** **Subjects’ questionnaires: questions (English translation; original in Italian) and answers**

**Question**	**Pre**		**Post**	
	**Correct**	**Wrong**	**Correct**	**Wrong**
Where should you walk?	4	2	6	0
Where do you have to cross?	5	1	6	0
The traffic light is red. What do you do in this situation?	3	3	5	1
The traffic light is yellow. What do you do in this situation?	3	3	5	1
The traffic light is green. What do you do in this situation?	5	1	5	1
What should you do if a traffic light is not present or is not working?	6	0	6	0

### Parents’ and caregivers’ questionnaire

We administered a different questionnaire, focused on the transfer of the learned skills in the subjects’ everyday life, to both parents and caregivers. The questionnaire included six questions, and the answers were based on a 6-point Likert-type scale, ranging from ‘never’ (0) to ‘always’ (5); see Additional file 2 for details. This is an interval scale, and parametric testing is justified provided that the normality assumptions cannot be rejected. To assess the effect of treatment separately for each individual question and on the cumulative subjects’ score, we used either a paired-samples t-test or a Wilcoxon’s test depending on the normality test (Lilliefors).

We also looked at the correlation between the answers of parents and caregivers. Significance was assessed by using the t-test for correlation.

## Results

Most subjects had little difficulty in learning the vocabulary of gestures required to interact with the VE. Subject S7 was not able to complete the familiarization phase because of problems with depth perception within the VE. Consequently, he was excluded from the treatment phase of the study. All six remaining subjects completed the whole experimental protocol. Three subjects (S2, S3, S4) – including the two with intellectual disability, S2 and S3 – could not complete the task at T0, but eventually achieved this ability by the end of the treatment phase (T1). All the subsequent analysis is based on the six subjects that completed the whole study protocol.

### Number of errors

**Figure 3:**
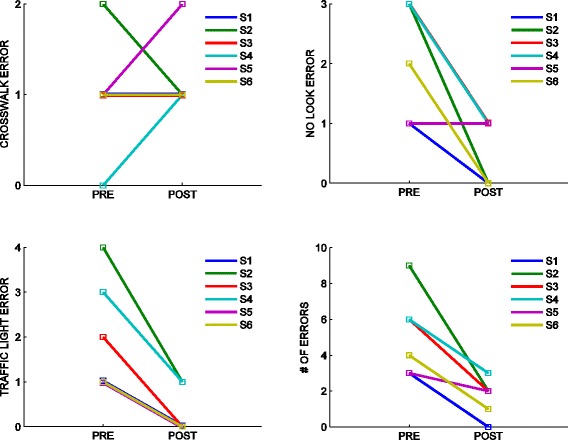
**Changes in performance errors from before (T0) and after treatment (T1).** Top: crosswalk errors (left), No look errors (right). Bottom: Traffic light errors (left), Total errors (right). Individual subjects are denoted by different colors.

The normality hypothesis had to be rejected (p > 0.05; Lilliefors test). We therefore used a Wilcoxon’s sign test to assess the treatment effect. When comparing the number of errors at T0 and T1, we only observed a significant decrease in the ‘crossing with red/yellow light’ error (p = 0.0313; Wilcoxon’s sign test). No significant changes were observed in the other error types. The change in the total number of errors was equally not significant (Wilcoxon’s sign test); see Figure 3.

### Navigation performance

**Figure 4:**
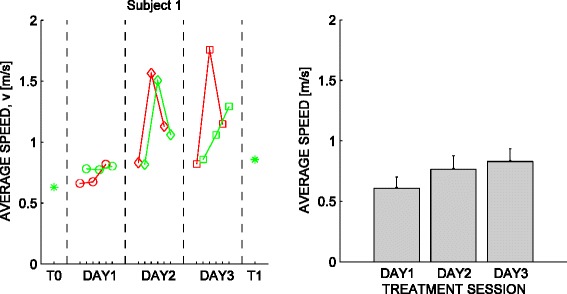
**Effect of training on the average speed.** Left: typical subject (S1). Red and green lines denote, respectively, Path A and Path B. Right: average over subjects, repetitions and paths.

**Figure 5:**
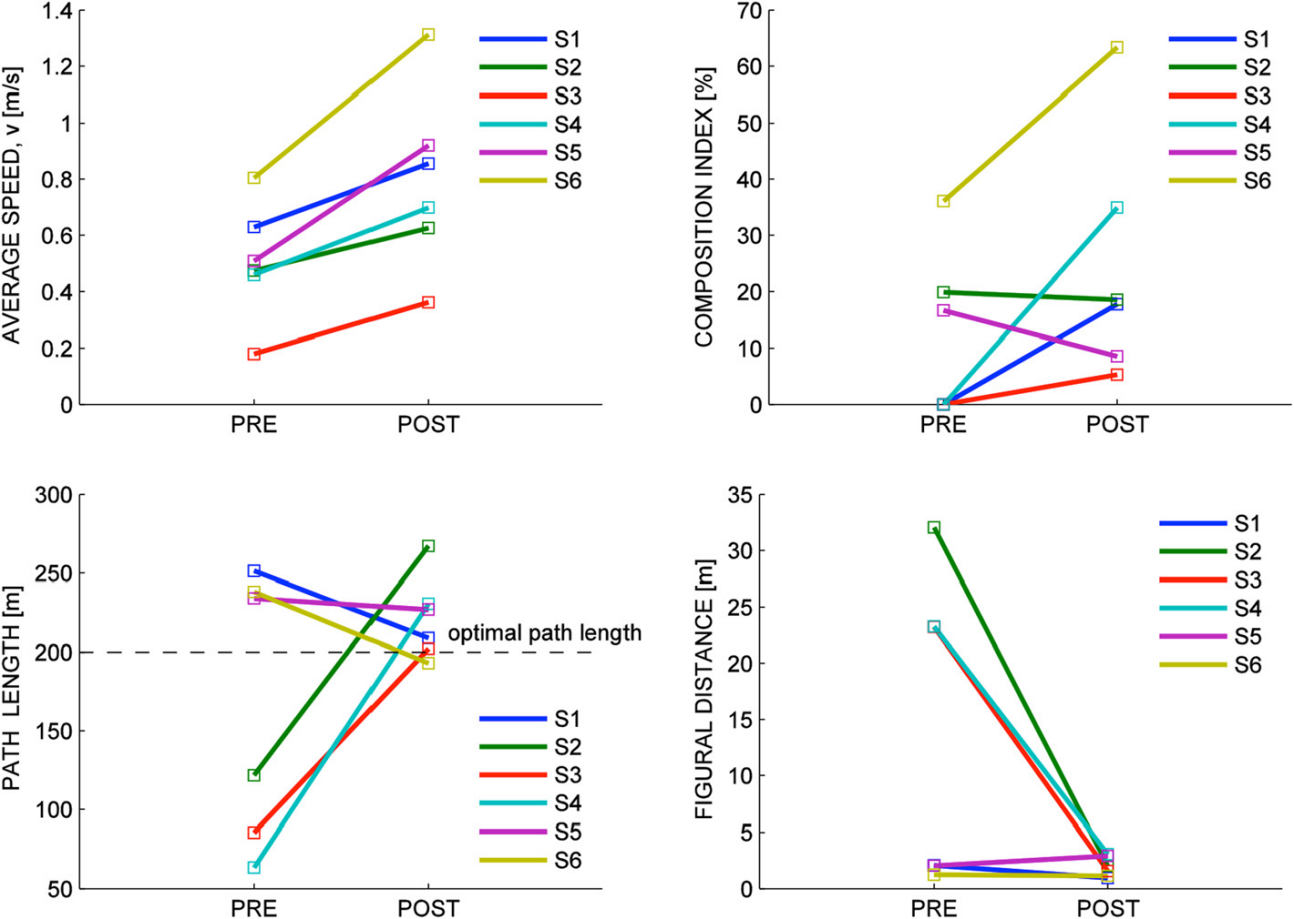
**Changes in navigation performance before (T0) and after treatment (T1).** Top: average speed (left) and composition index (right). Bottom: Path length (left) and figural distance (right). Individual subjects are denoted by different colors.

The effect of training on the average navigation speed is displayed in Figure 4. The normality assumption could not be rejected for these indicators. When looking at the ability to follow the street signs, we found that subjects significantly increased (p = 0.0042; paired-samples t-test) their average speed from T0 to T1, of an amount ranging from 40% to 100%; see Figure 5, top.

We found no significant changes in path length, figural distance, and composition index. As regards the composition index (CI), 4/6 subjects exhibited an increase from T0 and T1 – indicating that they became increasingly proficient in making multiple movements at the same time, e.g. foot forward and abduction of the arm. In the two remaining subjects (S2 and S5), the CI either decreased or remained almost constant. However, their CI was already significantly greater than 0 before training; see Figure 5, top.

Path length and figural distance - see Figure 5, bottom – revealed two typologies of behaviors. A group of subjects (S1, S5, S6) could complete the whole path from the very beginning (large – greater than optimal - path length and small figural distance). After training, their trajectories became closer to optimal (near-optimal path length, lower figural distance). The other group of subjects (S2, S3, S4) were initially unable to complete the path (low path length, high figural distance), but were able to do it after treatment (path length increase, figural distance decrease).

### Questionnaires: subjects

**Figure 6:**
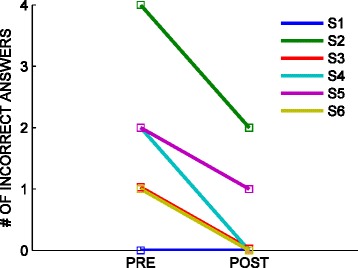
**Number of errors (incorrect answers) in the subjects’ questionnaire before and after treatment.**

The normality hypothesis had to be rejected (p > 0.05; Lilliefors test) so we turned to non-parametric statistics. A comparison of the subjects’ pre- and post-treatment answers to the questionnaire showed no statistically significant decrease in either the wrong answers to individual questions or the total number of errors (Wilcoxon’s signed-rank test for matched pairs), thus suggesting that subjects did not exhibit a significant improvement in their understanding of the task; see Figure 6.

The overall fraction of incorrect answers decreased from 28% (pre-treatment) to 8% (post-treatment); see Table 3.

We found no statistically significant changes when looking at the individual answers.

### Questionnaires: parents and caregivers

**Figure 7:**
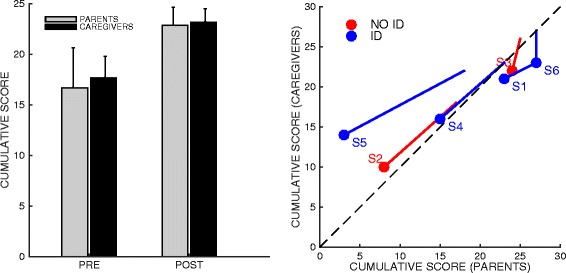
**Cumulative score of the parents’ and caregivers’ questionnaire, before and after treatment (left) and correlation between the two scores (right).** The filled circles denote the initial (start) score; the lines indicate the final score (end). Red and blue lines denote subjects with and without intellectual disability (ID).

As regards the questionnaires completed by parents and caregivers, the normality assumption could not be rejected. The changes in the total scores were significant for both parents (p = 0.0438; paired-samples t-test) and caregivers (p = 0.0030; paired-samples t-test); see Figure 7.

No statistically significant changes were observed when looking at the individual answers.

We also looked at the correlation between the answers of parents and caregivers. We found a significant correlation for both PRE and POST treatment answers (respectively, r = 0.9127; p = 0.011 and r = 0.8148; p = 0.0483), which suggests a substantial agreement between parents and caregivers in evaluating the subjects’ street crossing skills.

### Stereotypical behaviors

Several subjects exhibited stereotypical behaviors before treatment. S1 repeated sentences heard in the movies; S2 pounded his hand on his chest; S4 emitted small cries and waved his hands. Although we could not quantify the effect, we observed a substantial reduction or even disappearance of these behaviors during training.

## Discussion

The primary goal of this study was to investigate whether an integrated approach based on virtual environments and natural interfaces could be used to teach safety skills in adults with ASD, either with or without intellectual disability.

The focus on adults with ASD and the use of a gesture-based form of interaction with the VE are distinctive aspects of this study. Initially developed for gaming, gesture-based interfaces are increasingly used for both physical and cognitive rehabilitation purposes, including ASD [29,30]. We specifically assessed whether the proposed method and apparatus is useful in facilitating the acquisition of two specific skills: (i) crossing a street at the right time and in the proper way; and (ii) following street signs in order to reach a specific destination.

### The proposed apparatus and method was well accepted by all participants

No participant refused to use the system. Subjects were often tired at the end of the experimental sessions, but they always promptly agreed to come back for the next session. This is important because it may be hard to convince ASD subjects to do something that they do not like (American Psychiatric Association 2013). Anedoctally, we further observed that our subjects not only gladly agreed to use the system, but they actually enjoyed it.

Frequent refusals have been reported when goggles and headsets mediated the interaction with the VE. Persons with ASD often refuse to use these devices, possibly because they do not tolerate well the associated sensory over-stimulation [38]. Natural interfaces are expected to mitigate this. Also, they do not require a physical contact with the subjects’ body. Therefore, they are expected to be much more acceptable by ASD subjects. Our results confirm this prediction and are in agreement with [30], who used natural interfaces in an augmented reality system to train ASD individuals to match target body postures. Several studies have addressed the limitations of the Kinect system as a motion capture device in terms of accuracy, resolution, repeatability, delay. However, in this study the Kinect system is only used as a user interface, based on a very simple method for gesture recognition for which high accuracy is less crucial.

### ASD subjects learned to use the gesture-based interface for interacting with a virtual environment

In order to interact with the virtual environment, subjects had to learn a vocabulary of body gestures. The choice of these gestures is clearly a major determinant of ease of interaction and ultimately learning performance. The gesture vocabulary used here was selected through a series of preliminary tests that involved both healthy subjects and ASD subjects, in which we looked at ease of control (but did not quantify this from a psychometric standpoint) and ecological aspects (similarity to actual pedestrian movements). We cannot say that we ended up with the best possible interaction modality, but we are quite confident that it serves its purpose.

Subjects learned by imitation, by observing the therapist who showed them the vocabulary of body gestures. Most subjects easily acquired this ability; only one subject (S7) had problems with the interface, mostly due to a difficulty to perceive the depth dimension through the projection screen. For this reason this subject was unable to complete the familiarization phase and was therefore excluded from the study. Impaired depth perception has been previously reported in some ASD subjects [39]; see [40] for review. This type of perceptual impairment was not assessed at the enrollment, and our results suggest that it should be an exclusion criterion. All other subjects learned the simple body gestures required to interact with the VE. Subjects with intellectual disability initially had more difficulty, but they finally managed to complete the task just like the participants with no intellectual disability. This is in agreement with [21].

During training, in all participants we observed a decrease in stereotypical movements. This is consistent with [41], who reported that the stereotypical body movements in children with ASD decreased significantly during interaction with the VE.

We also observed an improved attention to the different scenarios of the training protocol. The subjects consistently kept their eyes on the screen and were not distracted by the therapist or other objects in the room. These results confirm the observations of Eynon A [42] and Josman N, Ben-Chaim HM, Friedrich S and Weiss PL [21] that VE interaction stimulates the participants’ attention.

### Subjects successfully learned the ‘virtual’ version of the safety skill

One primary goal of the proposed training protocol was to teach ASD subjects the rules of behavior that need to be followed to safely cross a street in a city environment - with crosswalks, traffic lights, cars and presence of other people. In particular, subjects had to learn (i) to use the crosswalks to cross a street; (ii) to look left and right before crossing; and (iii) to wait until the traffic lights – if present - turned green.

To quantify whether they actually learned these rules of behavior, we looked at the number of errors made during training in the virtual environment. We only observed a significant decrease in one specific error type, namely ‘crossing with red or yellow light’. In contrast, no significant change was observed in the ‘walking outside the sidewalk or crosswalk’ and ‘crossing without looking’ errors. Apparently, subjects easily learned behaviors that are triggered by simple color stimuli, like ‘start crossing on green’, or ‘wait on red’. Instead, behaviors like ‘walk within the sidewalk or crosswalk’ were more difficult to learn, possibly because they implies an ability to analyze more complex scenarios, involving a broader variety of contexts. This is consistent with previous observations [21]. This difficulty may be related to the impaired ability of ASD subjects to deal with multiple actions at the same time [43].

From these observations alone, we cannot conclude that subjects actually learned to use good judgment, planning, problem solving with regard to the environment in which they are moving - skills that are usually deficient in persons with autism [4]. On the other hand, our findings cannot be simply explained in terms of a memorization of the complex sequence of actions necessary to follow the paths – which are not totally predictable as every time the traffic lights may be in a different state.

A comparison of the pre- and post-treatment answers to the subjects’ questionnaire suggests a trend toward a decrease in the number of errors. The latter cannot be simply explained in terms of memorization of the correct answers, because the study participants received no feedback after questionnaire administration. Similarly, it is unlikely that familiarization with the task played a role as its focus was on learning the gestures to navigate the VE, not the actual street crossing skills. Nevertheless, statistical analysis revealed no significant changes in either the individual answers or the total number of errors. This is not unexpected, given the small sample and the low statistical power of the non-parametric tests.

### Subjects improved their path following performance

The movement indicators provide information on the way subjects improved their path following performance. We found that all subjects increased their movement speed from pre- to post-treatment evaluation. The same significant increase in the movement speed was observed within each of the three days of treatment. Overall, this result suggests that subjects gradually became more effective in activating the executive functions necessary to perform the required actions to move within the virtual environment.

When looking at the distance travelled and at how the observed movements compare to the optimal path, we found that all subjects fall into two distinct categories. Some ‘high-performance’ subjects – namely, S1, S5, and S6 - were capable of achieving the goal from the very beginning. With training these subjects gradually optimized their path, by reducing its length and by getting closer to the optimal path. In contrast, ‘low performance’ subjects - S2, S3, and S4 - were initially unable to complete the task within the 10 min timeout. These subjects exhibited an initially low path length and a large figural distance. However, after training their performance was comparable to that of the high-performance group. We cannot speculate any further because of our limited sample, but the differences between these subjects groups cannot be simply explained in terms of IQ differences or in terms of presence/absence of speech – see Table 1.

Overall, these results confirm that interaction with a VE is effective in helping participants to acquire the ability to reach a certain place by following the street signs [19].

### Do the learned abilities generalize to real-life situations?

In real life situations, the application of safety skills often leaves little margin for errors. Our results do not suggest that all errors were eliminated. The questionnaire administered to the study participants before and after treatment failed to demonstrate that they actually improved their knowledge of the rules for moving safely in a city environment.

We did not directly test whether training had an impact in the subjects’ life. Rather, we administered a second questionnaire independently to the subjects’ parents and caregivers that aimed at assessing the transfer of the practiced skill in real life situations. Both parents and caregivers reported a significant post-treatment improvement in the subjects’ performance. They also provided highly consistent answers. These observations highlight the common perception of parents and caregivers that the behaviors acquired through training within the VE resulted in an improved attention when subjects engaged in similar behaviors (street crossing) in daily life situations.

However, these findings do not allow to conclude that VE training generalized to real-life situations. This is in fact a major limitation of this study. Our observations will need to be confirmed by larger study and by a more direct assessment of transfer.

## Conclusions

Persons with autism need a continuity of care, based on their individual needs and their degree of impairment, which both change with the evolution of their general health state and with their natural development. The international management guidelines [44] recommend highly specific and personalized treatments. The most common rehabilitative interventions are based on psycho-educational approaches that take into account both the skills of each individual and the characteristics of his living environment. The overall goal of these interventions is to improve their independence in activities of daily living, such as moving autonomously in an outside environment. ASD subjects learn best in structured environments through repeated practice of a stereotypical sequence of actions. There are no standardized rehabilitative methods for adults with autism; therapeutic intervention is based mainly on educational treatments that can facilitate the acquisition of safety skills, which improve their quality of life.

Our findings extend to adults with ASD a number of previous studies that involved children or adolescents [15,21].

The small number of subjects is a major limitation of this research and our observations will need to be confirmed by a larger study. Overall, the participants exhibited an high degree of acceptance of this form of exercise, and all promptly agreed to come back for the next session. The study outcomes indicate the feasibility of interacting with a VE through a natural interface to facilitate the acquisition of street safety skills by adults with ASD.

We only made an indirect assessment of transfer of the learned skills to real life. Also, we did not look at the long-term retention of the acquired rules. As noted by [11], these are common problems in treatments based on technologies that are subject to a rapid evolution: validation studies often cannot keep the pace with the fast rate of development of technological innovations, and transfer from VE practice to real-life is difficult to assess, specially for safety skills that leave little margin for errors.

The Kinect system is exemplary in this respect: low-cost motion capture systems have a still largely unexplored potential to revolutionize many fields in the rehabilitation domain, including education of persons with special needs. Until a few years ago, interaction with VE as a tool for rehabilitation – in cognitive and behavioral domains – was restricted to a few research centers. Instructors and therapists use VEs as educational and therapeutic tools to provide persons with ASD a safe environment for learning. The proposed method relies on a consumer motion capture device and open-source VR software, which makes the system particularly suitable for any educational and clinical setting.

## Additional files

Additional file 1:
**Questionnaire for subjects (English version; original in Italian).**
Additional file 2:
**Questionnaire for parents and caregivers (English version; original in Italian).**
