# Supplementary material for: Natural interfaces and virtual environments for the acquisition of street crossing and path following skills in adults with Autism Spectrum Disorders: a feasibility study
Source: J Neuroeng Rehabil. 2015 Feb 19;12:17. doi: 10.1186/s12984-015-0010-z (PMC4344805; doi:10.1186/s12984-015-0010-z)

1. Where do you have to walk?

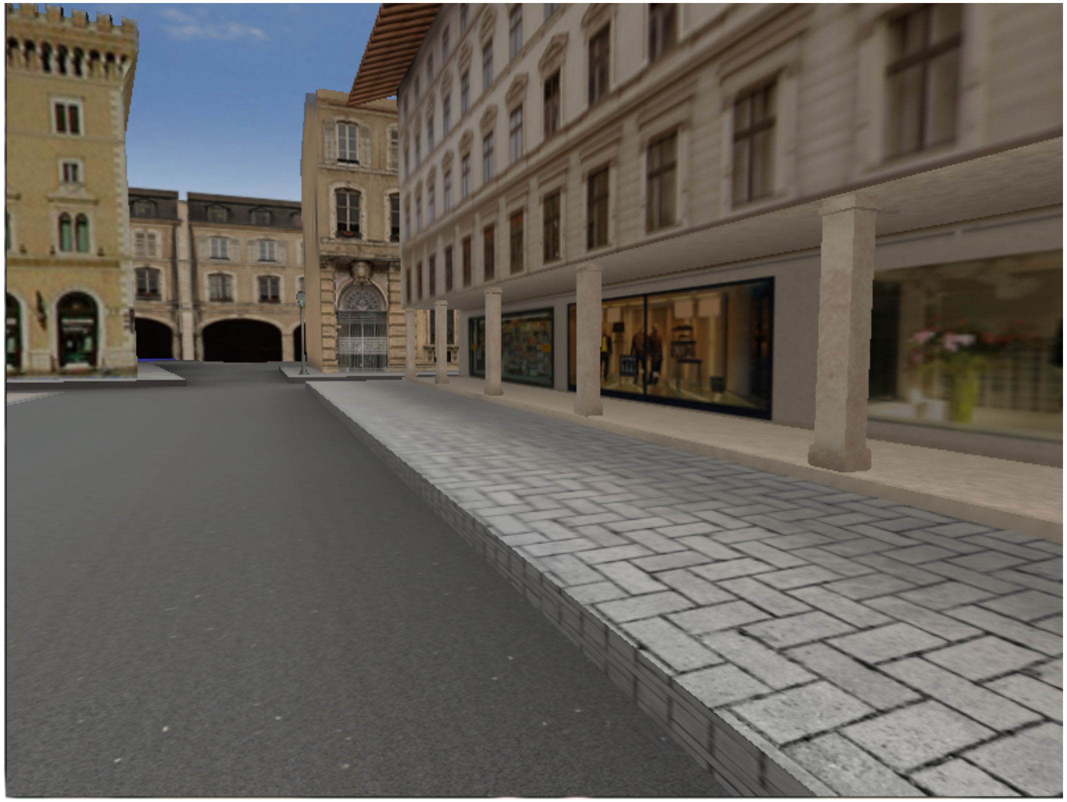

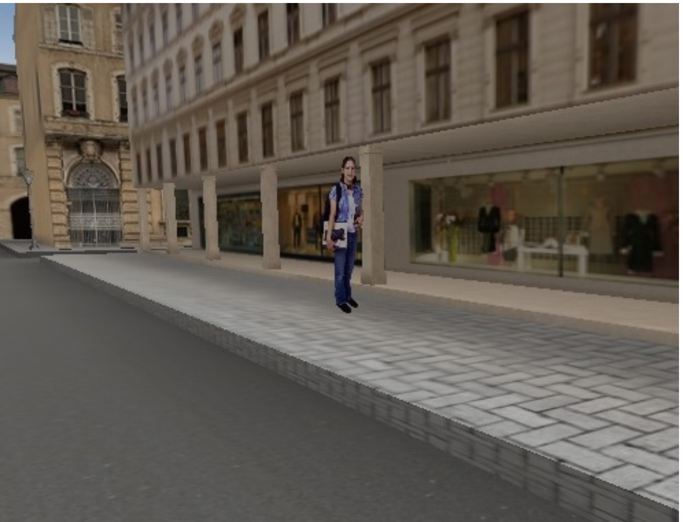

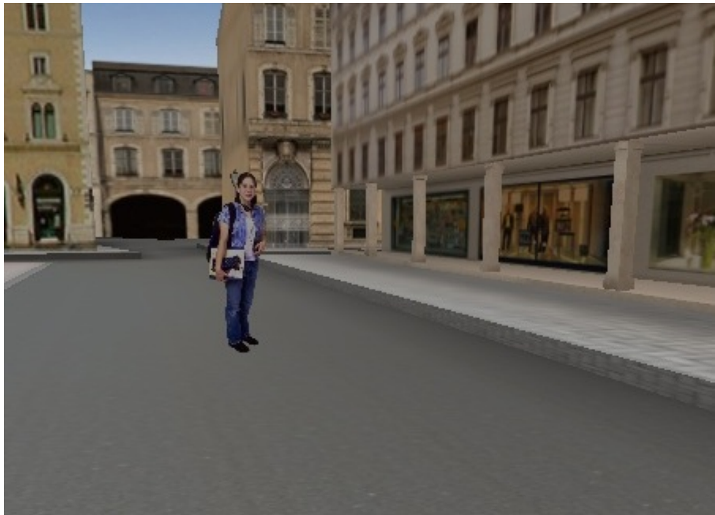

2. Where do you have to cross?

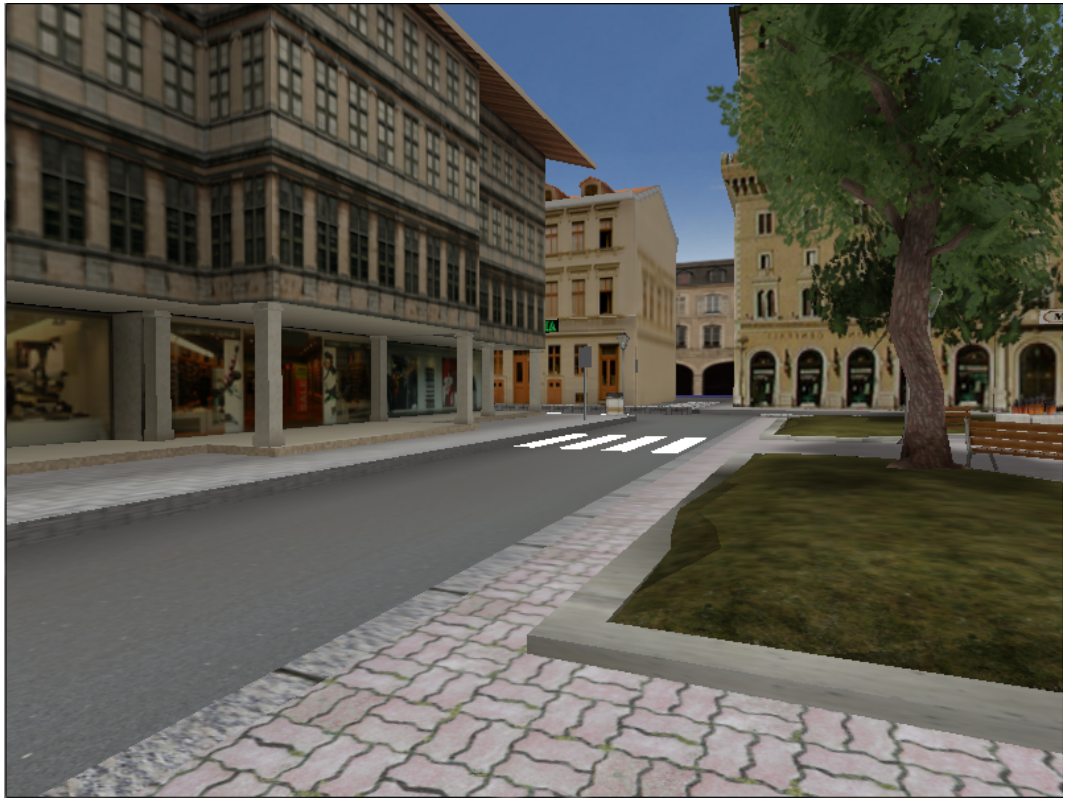

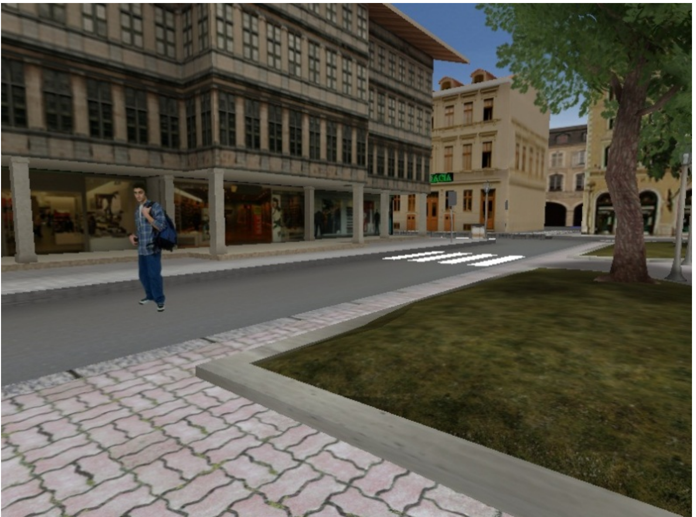

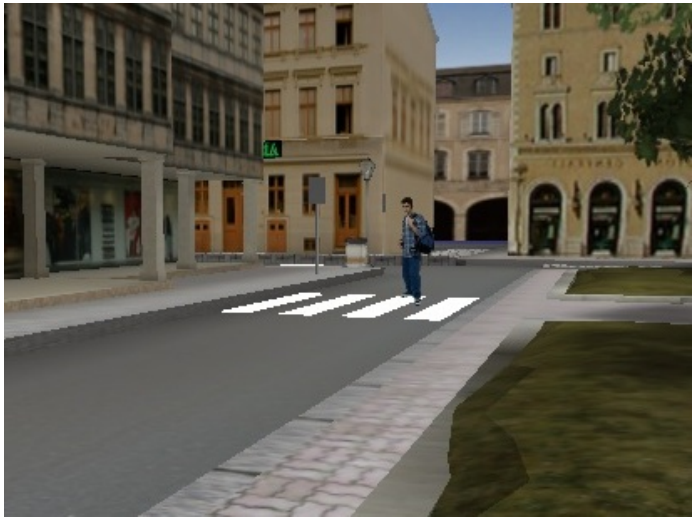

3. The traffic light is red. What do you do in this situation?

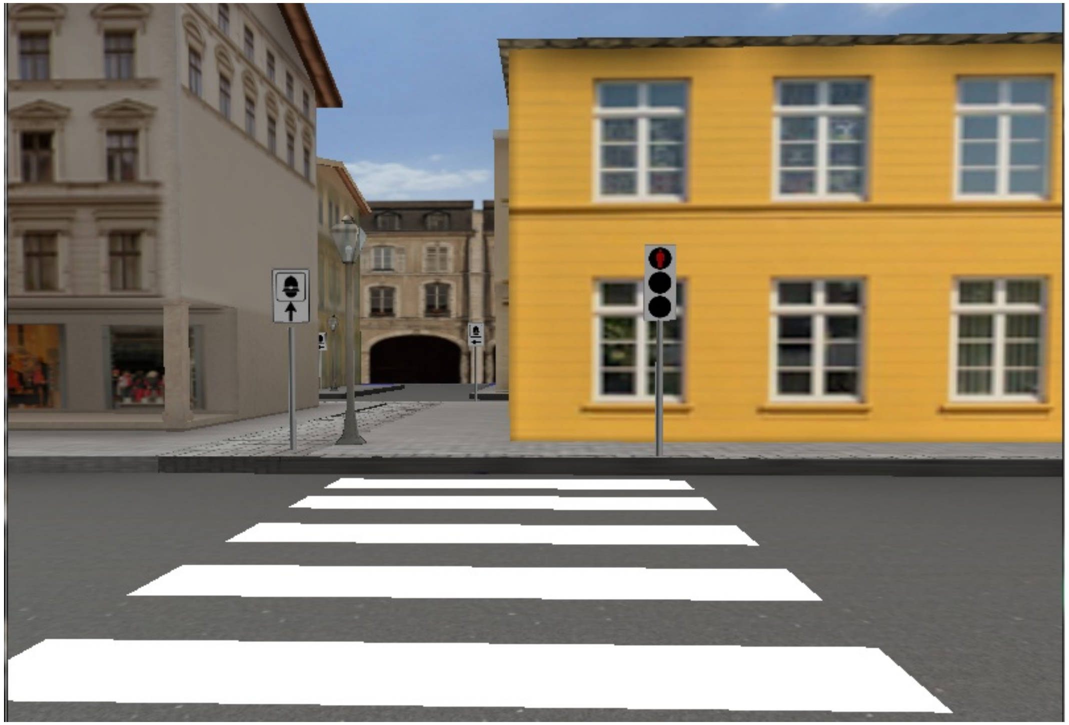

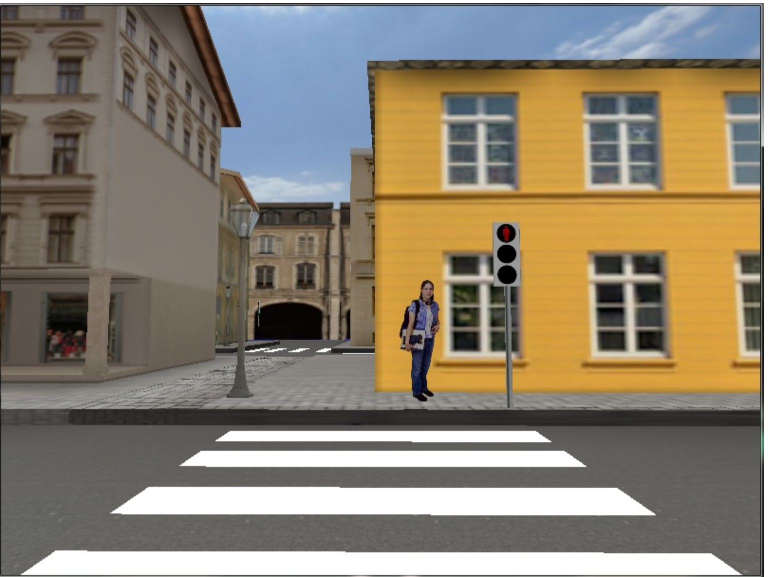

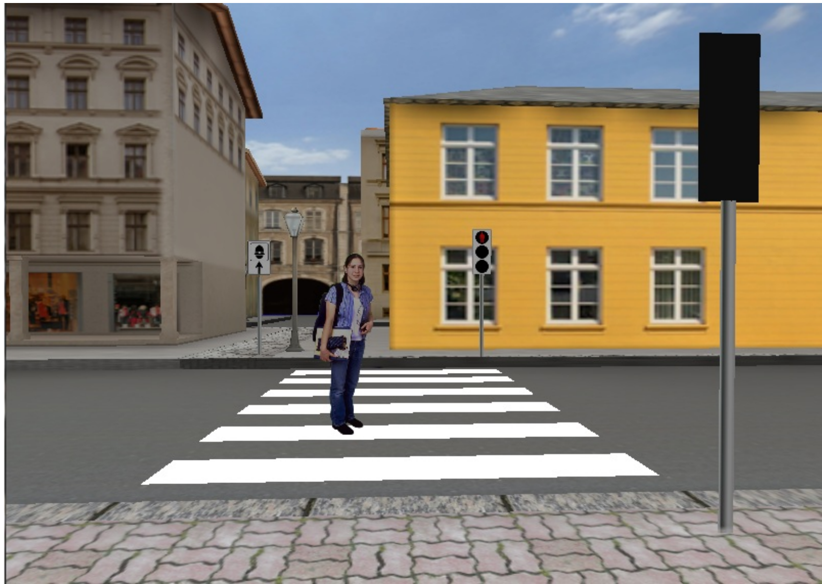

4. The traffic light is yellow. What do you do in this situation?

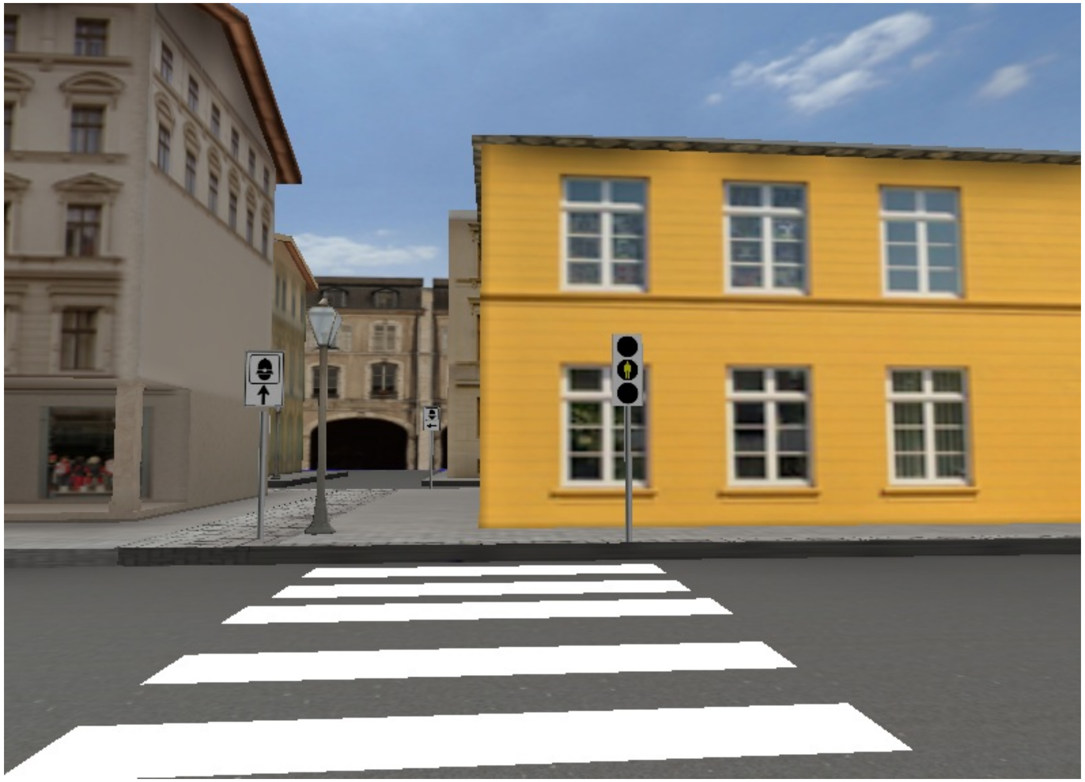

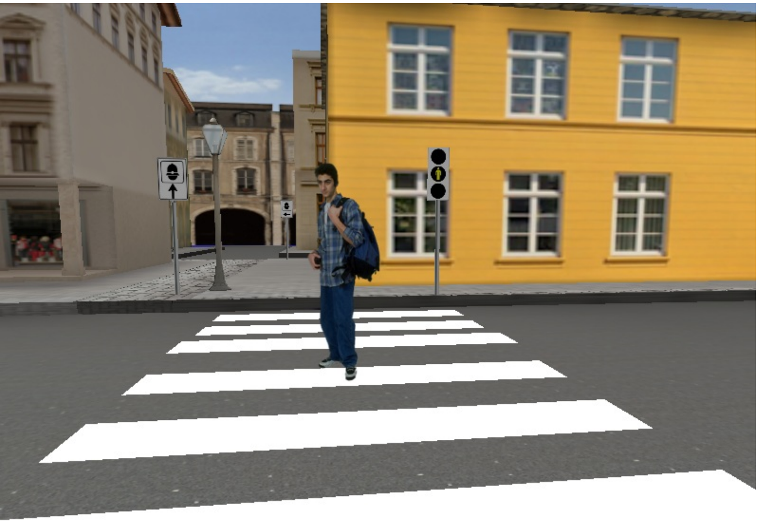

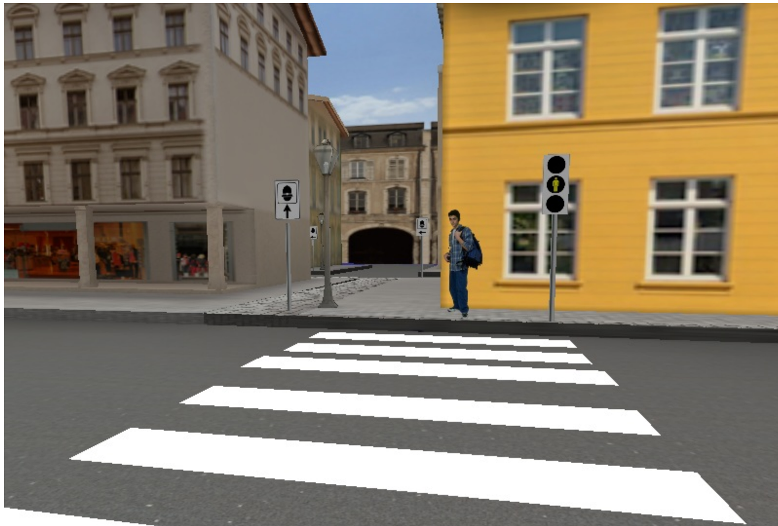

5. The traffic light is green. What do you do in this situation?

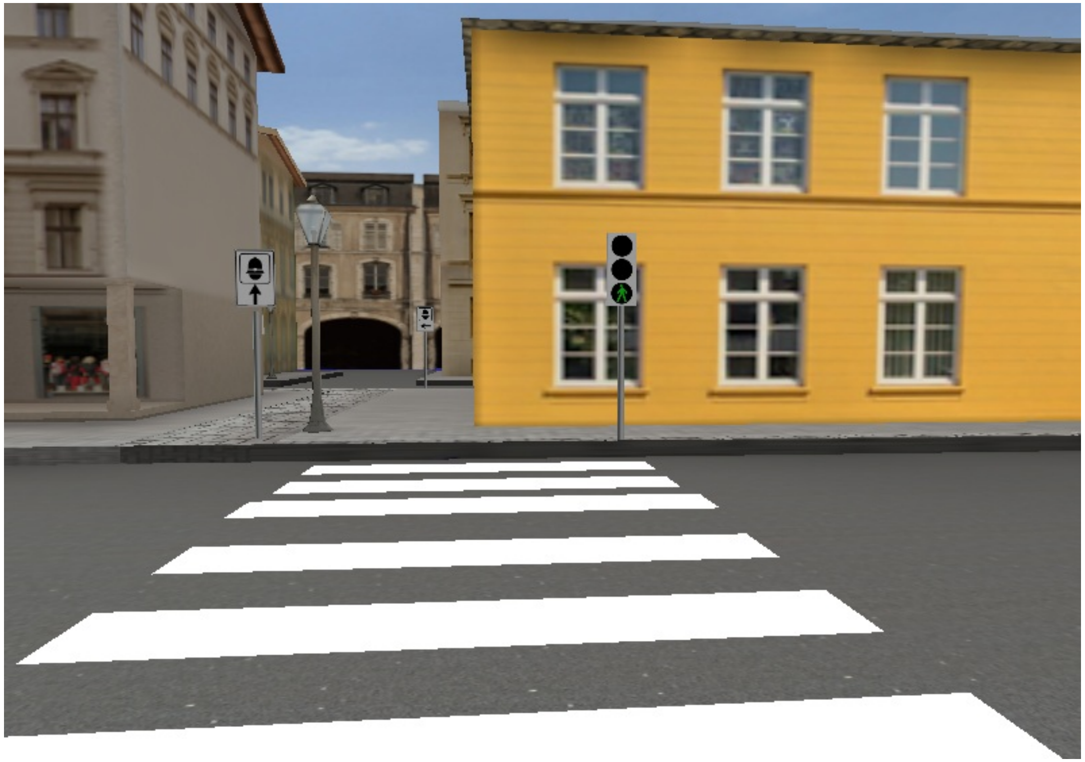

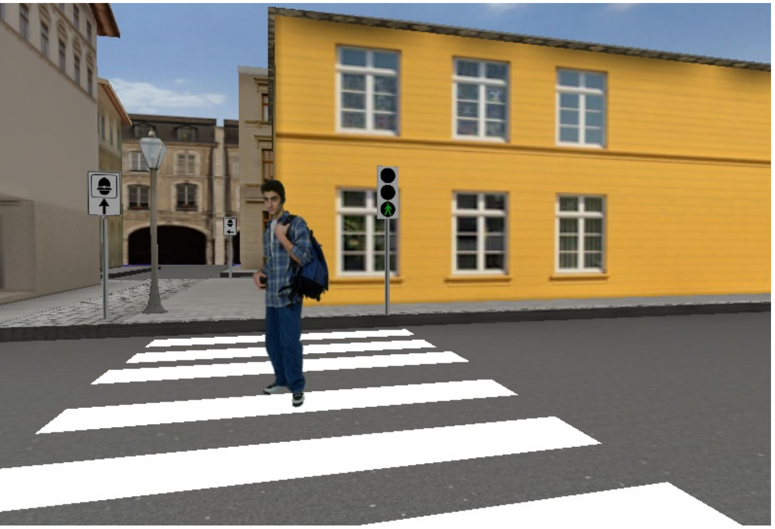

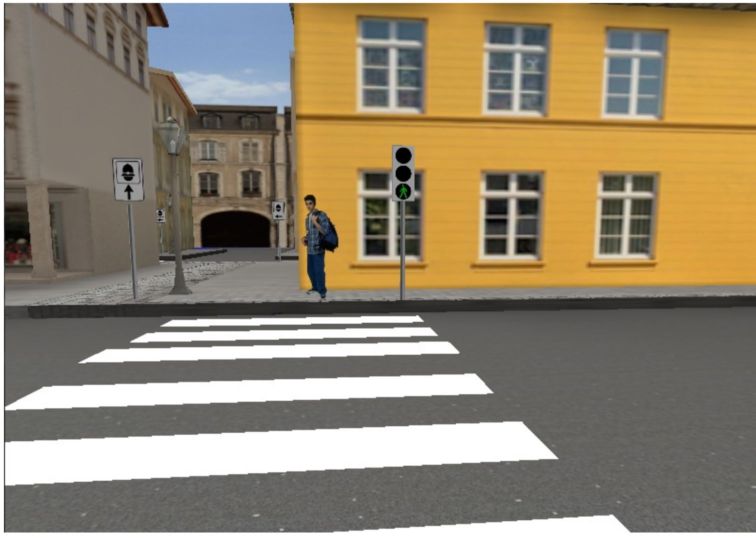

6. What should you do if a traffic light is not present or is not working?

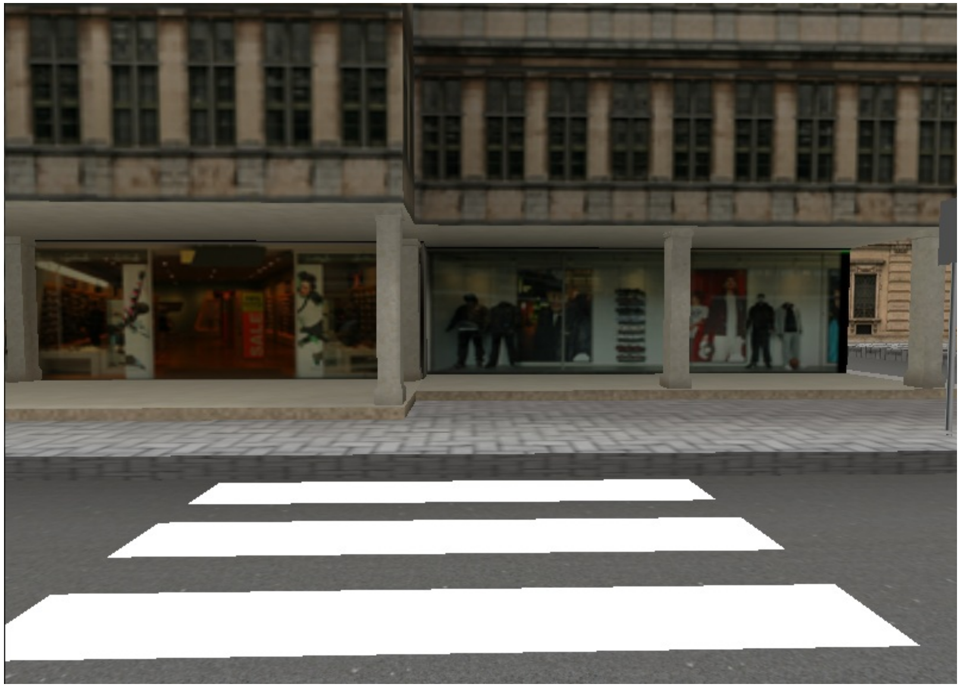

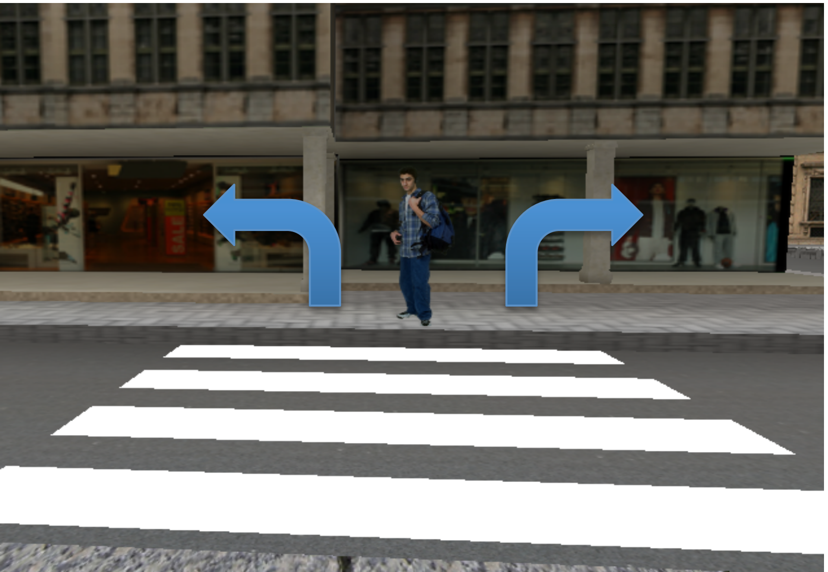

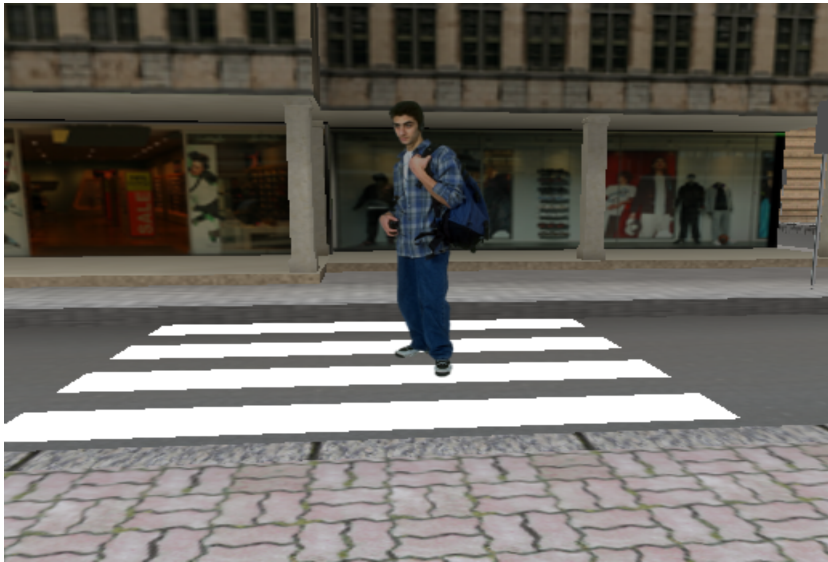

Supplement: Additional file 1: — Questionnaire for subjects (English version; original in Italian). [file 12984_2015_10_MOESM1_ESM.pdf]
