# Supplementary material for: Natural interfaces and virtual environments for the acquisition of street crossing and path following skills in adults with Autism Spectrum Disorders: a feasibility study
Source: J Neuroeng Rehabil. 2015 Feb 19;12:17. doi: 10.1186/s12984-015-0010-z (PMC4344805; doi:10.1186/s12984-015-0010-z)
Supplement: Additional file 2: — Questionnaire for parents and caregivers (English version; original in Italian). [file 12984_2015_10_MOESM2_ESM.pdf]

Subject	
Date	

- ☐ Parent
- ☐ Caregiver
- ☐ PRE-treatment
- ☐ POST-treatment

Can the subject identify where to walk when he/she is outside in the streets?

0	1	2	3	4	5

Can the subject identify where to cross when he/she is outside in the streets?

0	1	2	3	4	5

Does the subject wait when the traffic light is red?

0	1	2	3	4	5

Does the subject wait when the traffic light is yellow?

0	1	2	3	4	5

Does the subject cross when the traffic light is green?

0	1	2	3	4	5

When the traffic light is off, does the subject look to the right and to the left before crossing?

0	1	2	3	4	5

Legend

0= behavior absent
5=behavior is always present
